# Synergistic pretreatment: hybrid strategies for maximum lignocellulose valorization

**DOI:** 10.1007/s10532-026-10296-9

**Published:** 2026-04-22

**Authors:** Nikunj Mehta

**Affiliations:** School of Medicine and Public Health, University of Wisconsin-Madison, Madison, WI USA

**Keywords:** Lignocellulosic biomass, Pretreatment, Enzymatic saccharification, Recalcitrance, Biorefinery

## Abstract

Lignocellulosic biomass (LCB) is a vast, renewable resource critical to a circular bioeconomy, but its inherent recalcitrance remains the principal barrier to efficient enzymatic saccharification and valorization. Given the numerous existing reviews that simply catalogue individual pretreatment methods, the necessity of this review lies in its critical evaluation of how hybridizing standalone technologies is essential to overcome current pilot-scale and commercialization bottlenecks. This review provides a comparative analysis of three emerging pretreatment technologies: hydrothermal (HTP), microwave-assisted (MWP), and ball milling (BM). The author analyzes the distinct mechanisms by which each technology decreases the recalcitrance of LCB. HTP excels at hemicellulose hydrolysis via autohydrolysis but is plagued by the formation of inhibitors and pseudo-lignin. MWP employs rapid dielectric heating to achieve similar objectives within minutes, compared with the hours often required for conventional HTP, demonstrating high energy efficiency (e.g., 40.1 kJ/g compared to conventional HTP at 70.85 kJ/g), but faces fundamental commercial scale-up challenges related to finite penetration depths and hotspots. BM, a mechanochemical approach, is unparalleled in destroying cellulose crystallinity, dramatically enhancing kinetics without producing inhibitors, but it suffers from prohibitively high energy consumption, often requiring up to 2.8 kWh/kg. The author concludes that commercial viability dictates a trend toward hybrid, synergistic processes, such as BM-HTP and MW-HTP, which balance trade-offs and achieve near-theoretical glucose yields of 97.3%. Future research must focus on continuous-flow reactor engineering, integration with lignin-first valorization strategies, and predictive AI/ML modeling to enable economically competitive lignocellulosic biorefineries.

## Introduction

**Fig. 1:**
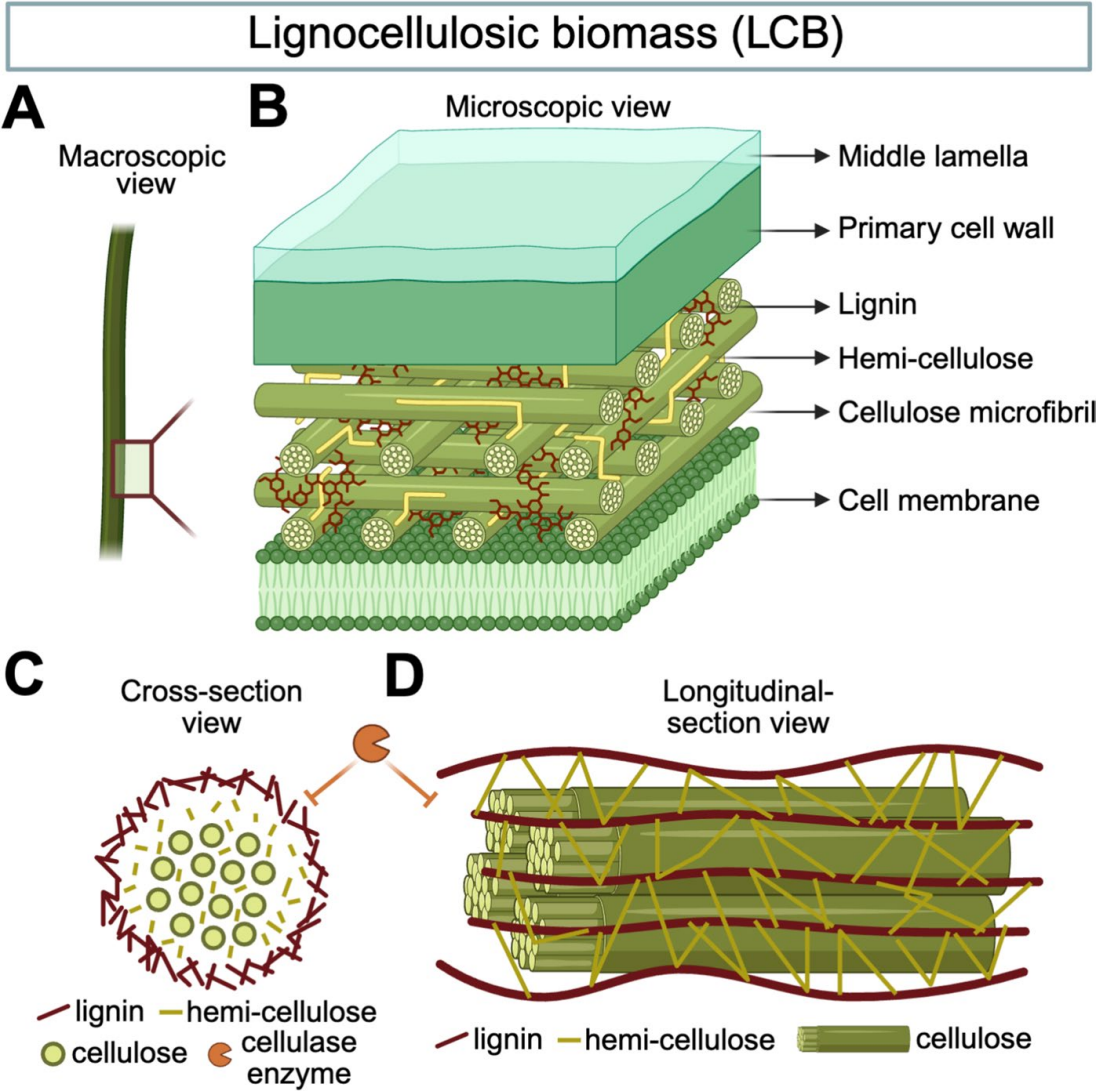
The multi-scale architecture of lignocellulose and its recalcitrance barrier. **A** The macroscopic view of raw lignocellulosic biomass (LCB) feedstock, derived from agricultural or forestry waste. **B** A microscopic, close-up view of the plant cell wall reveals a complex, heterogeneous composite structure. At the nanoscale molecular level, the key factors inhibiting efficient downstream enzymatic hydrolysis are depicted. Cellulose (40–60% of LCB) consists of highly ordered, crystalline microfibrils (Zoghlami and Paes 2019). These rigid, crystalline domains are tightly sheathed by amorphous hemicellulose (20–35% of LCB) (Zoghlami and Paes 2019) and are chemically cross-linked and encased by lignin (10–25% of LCB), an aromatic polymer that acts as an impervious glue (Zoghlami and Paes 2019; Tanis et al. 2023). **C, D** Cross-section and longitudinal-section view, respectively, of the LCB, illustrating the LCB architecture consisting of cellulose, hemicellulose, and cellulose. This interconnected structure dramatically reduces the accessible surface area (ASA) (Zoghlami and Paes 2019) and physically shields the cellulose chain, effectively blocking the penetration and action of cellulase enzymes (orange) (Broda et al. 2022; Chen et al. 2018a). Overcoming this structural barrier is the primary goal of pretreatment. Images were created using BioRender

The transition toward a sustainable, circular bioeconomy is increasingly dependent on the development of advanced biorefineries capable of converting renewable resources into fuels, chemicals, and materials (At et al. 2020; Longati et al. 2018; Liu and Bao 2017). Lignocellulosic biomass (LCB), which comprises agricultural residues, forestry waste, and dedicated energy crops (depicting a macroscopic view of a plant stem in Fig. 1A), represents the most abundant and low-cost renewable carbon source on Earth (Banu, et al. 2021; Singh et al. 2022). Its utilization for producing second-generation biofuels and bio-based chemicals is essential for mitigating climate change and reducing dependence on fossil resources without compromising global food security (Zoghlami and Paes 2019; Kuglarz et al. 2018; Alio et al. 2020). However, the efficient valorization of LCB is severely hampered by its inherent recalcitrance to deconstruction (Zoghlami and Paes 2019). This recalcitrance is a complex, multi-scale defense mechanism evolved by plants to resist natural environmental stresses and microbial attacks, which consequently makes the biomass highly resistant to industrial mechanical, chemical, and biological treatments (Silveira et al. 2013). The deconstruction of this recalcitrant matrix is widely recognized as the most significant techno-economic bottleneck in the entire conversion process (Longati et al. 2018; Broda et al. 2022).

This recalcitrance arises from a combination of chemical and structural factors (Zoghlami and Paes 2019). Chemically, LCB is composed of three primary polymers: cellulose, a linear polymer of β-(1,4)-linked D-glucose units, organized into highly crystalline microfibrils that are resistant to hydrolysis and comprise 40–60% of the plant’s biomass (Zoghlami and Paes 2019); hemicellulose (20–35%), which is a heterogeneous, amorphous polymer (such as xylan and mannan) that encases the cellulose microfibrils (Zoghlami and Paes 2019), and lignin (10–25%), a complex, hydrophobic aromatic polymer that acts as a glue, cross-linking the polysaccharide components and providing structural integrity (Fig. 1B) (Zoghlami and Paes 2019). These components are extensively cross-linked, forming lignin-carbohydrate complexes (LCCs) that further shield cellulose (Tanis et al. 2023). Structurally, this chemical architecture results in low accessible surface area (ASA), limited pore volume, and a high degree of cellulose crystallinity, which together block enzyme access physically (Fig. 1C, D) (Zoghlami and Paes 2019).

Furthermore, the selection of a specific pretreatment method is fundamentally dictated by its subsequent downstream application (Broda et al. 2022; Olatunji et al. 2021). For instance, the production of bioethanol (EtOH) requires highly accessible cellulose and is highly sensitive to furanic and phenolic inhibitors, thereby often favoring milder pretreatments, integrated/hybrid schemes, or processes coupled with effective detoxification steps (Broda et al. 2022; Ujor and Okonkwo 2022; Jilani and Olson 2023; Llano et al. 2021). Conversely, anaerobic digestion for biogas or dark fermentation for bio-H_2_ may, in some cases, accommodate certain pretreatment-derived compounds more effectively than yeast-based ethanol fermentation, although both remain susceptible to inhibition at elevated concentrations; therefore, pretreatment severity must still be carefully optimized (Olatunji et al. 2021; Jr and Kg 2021; Wang et al. 2024a; Zhao et al. 2025). Thus, understanding the intended end-use is critical for optimizing the techno-economic balance of the chosen pretreatment method (Jilani and Olson 2023; Llano et al. 2021; Zhao et al. 2025; Chakraborty et al. 2025; Kumar et al. 2025).

It is also important to recognize that pretreatment performance is inherently feedstock-specific (Raheja et al. 2024). Agricultural residues and forest-derived biomasses differ substantially in polymer composition and cell-wall organization, and these differences directly affect their response to pretreatment and subsequent enzymatic saccharification (Raheja et al. 2024; Woźniak et al. 2025; Segers, et al. 2024; Bertacchi et al. 2021). For example, softwoods are generally more recalcitrant than hardwoods, grasses, and many energy crops because they contain predominantly guaiacyl-rich, more condensed lignin and therefore often require higher pretreatment severity to achieve efficient cellulose hydrolysis(Segers, et al. 2024; Suota et al. 2021; Hossain et al. 2019). Even among agricultural residues, the same pretreatment does not produce uniform results: comparative work on rice straw, cotton stalk, and mustard stalk showed that dilute acid and steam explosion produced different extents of xylan removal and downstream glucose release depending on the biomass type, reflecting differences in structural recalcitrance (Gaur et al. 2015). Consequently, no single pretreatment can be considered universally optimal, and pretreatment selection and severity should be tailored to the specific feedstock and intended downstream application (Woźniak et al. 2025; Bertacchi et al. 2021).

The pretreatment of LCB is, therefore, the pivotal step for unlocking its recalcitrant structural matrix in the biorefinery (Namboonlue et al. 2025). Its primary goal is not to hydrolyze cellulose, but to disrupt the recalcitrant matrix, remove hemicellulose and lignin, and increase the accessibility of cellulose to enzymatic attack during the subsequent saccharification stage (Chen et al. 2018a). The efficiency of enzymatic saccharification, itself a costly step (Broda et al. 2022), is almost entirely contingent on the success of the pretreatment. The factors contributing to recalcitrance are deeply interconnected (Zoghlami and Paes 2019). Consequently, pretreatment is a multi-variable optimization problem. A method that aggressively targets one factor, such as hemicellulose removal, may inadvertently exacerbate another. For example, severe conditions can lead to the formation of inhibitory compounds or the reprecipitation of modified lignin, known as pseudo-lignin, which blocks enzyme access (Li et al. 2014). An ideal pretreatment must therefore provide a holistic solution that targets multiple recalcitrance factors simultaneously, a challenge that has driven the development of the technologies discussed in this review.

Recent literature from the last three to five years suggests a persistent synthesis gap: many reviews continue to emphasize pretreatment chemistries, feedstock-specific performance, or method-by-method comparisons, whereas comparatively fewer focus explicitly on industrial scale-up, operational stability, and techno-economic trade-offs (Woźniak et al. 2025; Kululo et al. 2025; Saad and Gonçalves 2024; Patel et al. 2025). This review provides a critical evaluation of three distinct and emerging pretreatment technologies: hydrothermal pretreatment (HTP), microwave-assisted pretreatment (MWP), and ball milling (BM). These three specific techniques were chosen because they represent the leading edge of thermal, electromagnetic, and mechanical process intensification, respectively. By comparing these distinct mechanisms, the review aims to highlight how the limitations of singular approaches necessitate synergistic integration. Furthermore, to bridge the gap between laboratory success and commercial viability, this review addresses the current state of early-stage techno-economic assessments (eTEA) and the emerging scope of artificial intelligence and machine learning (AI/ML) in optimizing these methods for pilot-scale implementation.

## Literature search methodology

To ensure a comprehensive and transparent evaluation of the selected pretreatment technologies, a systematic literature search was conducted. Databases including Web of Science, Scopus, PubMed, and Google Scholar were queried using specific search terms such as lignocellulosic biomass, hydrothermal pretreatment, microwave-assisted pretreatment, ball milling, enzymatic saccharification, and techno-economic analysis. The primary focus was on peer-reviewed articles published within the last five years to capture the most current state-of-the-art advancements, with foundational seminal papers included where necessary to explain fundamental mechanisms. Inclusion criteria prioritized studies that provided quantitative data on sugar yields, inhibitor formation, energy consumption, and scalability challenges.

## Hydrothermal pretreatment mechanisms and products

**Fig. 2:**
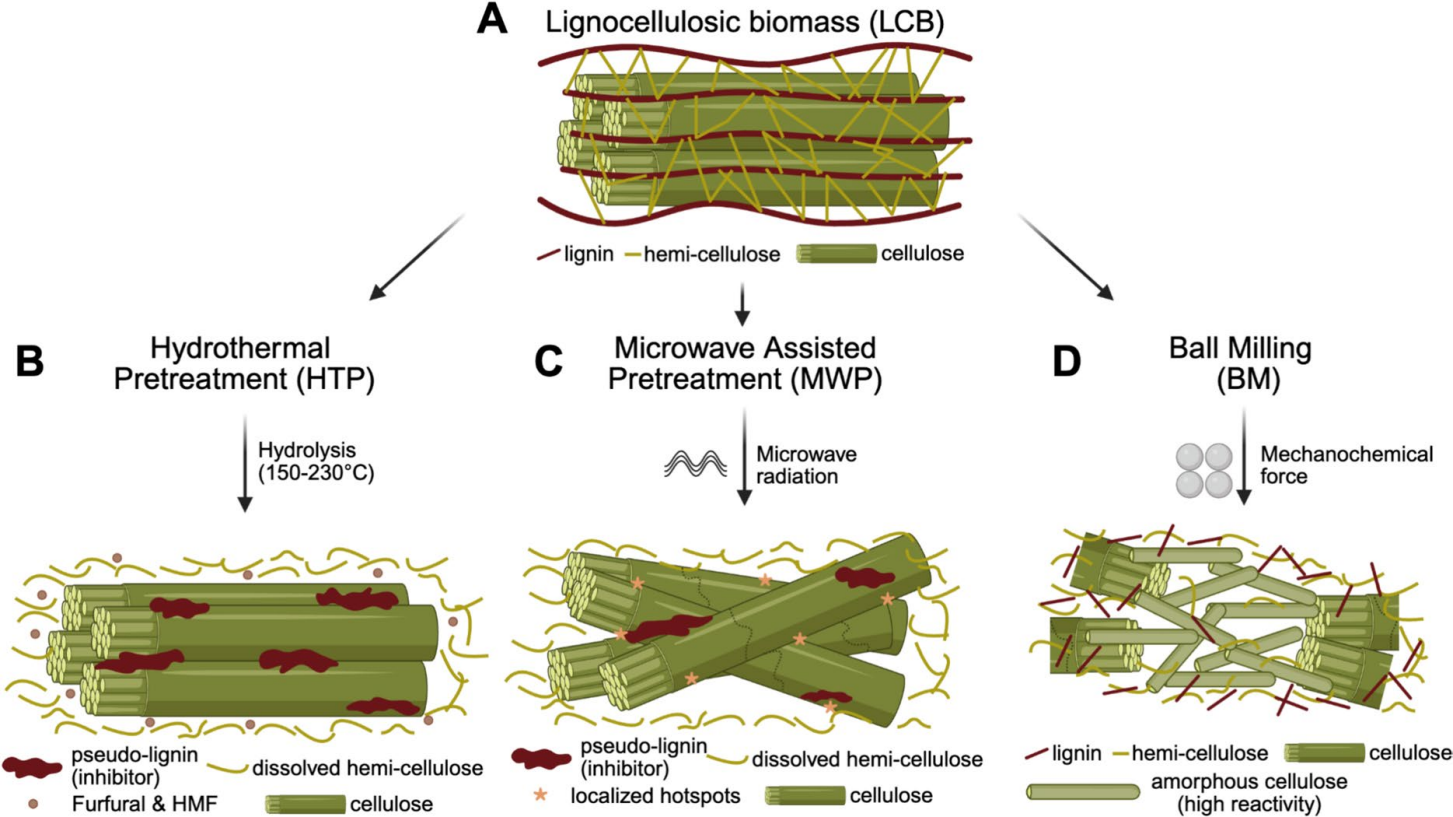
Schematic illustration of the distinct mechanisms by which hydrothermal pretreatment (HTP), microwave-assisted pretreatment (MWP), and ball milling (BM) reduce lignocellulosic biomass recalcitrance. This schematic illustrates the distinct primary mechanisms and immediate consequences of the three targeted pretreatment classes on the lignocellulosic matrix, in **A**. **B** HTP mainly promotes autohydrolytic solubilization of hemicellulose and disruption of lignin–carbohydrate linkages (Martín et al. 2022a; Ma et al. 2025), thereby exposing cellulose, although excessive severity may also generate inhibitors and pseudo-lignin(Martín et al. 2022a; Li et al. 2018). **C** MWP applies rapid volumetric heating, accelerating chemically assisted reactions through the oscillatory motion of polar molecules. This localized, selective heating generates hotspots that trigger intraparticle micro-explosions (Venegas-Vásconez et al. 2025), physically disrupting the cell wall matrix and weakening lignin-carbohydrate linkages(Venegas-Vásconez et al. 2025; Ethaib 2024). The rapid nature of the heating is advantageous as it minimizes reaction time, generally resulting in lower concentrations of chemical inhibitors compared to conventional batch HTP(Shukla et al. 2023; Kumar and Sharma 2017). **D** BM disrupts biomass physically by reducing particle size, increasing specific surface area, and decreasing cellulose crystallinity (Pérez-Merchán et al. 2022; Wilkinson et al. 2015). Its unique action is the physical destruction of the crystalline structure of cellulose (Yu and Wu 2011), converting the rigid cellulose I into a highly disordered amorphous state or, under aqueous conditions, to the more accessible Cellulose II allomorph (Lan et al. 2022). This dramatic internal structural change greatly improves enzyme kinetics. Crucially, as a purely physical process, BM produces negligible or zero chemical inhibitors (Baruah, et al. 2018). Images were created using BioRender

Hydrothermal pretreatment (HTP), also referred to as autohydrolysis, or subcritical water treatment, is a leading sugar-platform technology because of its relative simplicity and effectiveness (Martín et al. 2022a). Typically, it utilizes water at elevated temperatures (150–230 °C) and corresponding saturation pressures to maintain a liquid phase (Ruiz et al. 2020). The primary chemical mechanism is autohydrolysis (Fig. 2B). The process is autocatalyzed by the in situ formation of hydronium ions from water autoionization and organic acids, such as acetic acid, which are released from the deacetylation of hemicellulose acetyl groups (Martín et al. 2022a; Zhang et al. 2018a). These acidic conditions preferentially cleave the glycosidic bonds in the hemicellulose fraction.

Two major modalities of HTP discussed here are Liquid Hot Water (LHW) extraction and Steam Explosion (SE) (TR, Sarker., et al. 2021; WH, C., et al. 2022). LHW maintains biomass in a pressurized liquid aqueous environment and relies primarily on autohydrolytic solubilization of hemicellulose and modification of lignin–carbohydrate linkages; depending on severity, residence times typically span tens of minutes to hours (WH, Chen., et al. 2022; Jimenez-Gutierrez et al. 2021; Serna-Loaiza, et al. 2022). Because LHW does not include an abrupt depressurization step, its effects are predominantly chemical and structural rather than strongly mechanical, and it does not produce the rapid defibrillation and particle-size reduction characteristic of SE (WH, Chen., et al. 2022; Jimenez-Gutierrez et al. 2021; Tian et al. 2019). In contrast, SE comprises two distinct stages: steam cracking followed by explosive decompression, in which high-pressure steam rapidly heats the biomass and sudden pressure release causes flash evaporation of superheated water within biomass pores, thereby disrupting the fiber structure (Ziegler-Devin et al. 2021; T, Pielhop, et al. 2016; AT, Hoang., et al. 2023; NN, Idris., et al. 2025). Consequently, SE is generally completed on the order of minutes rather than hours and combines autohydrolytic chemistry with a pronounced mechanical effect that can reduce particle size rapidly; it has also been reported to require less energy for comparable size reduction than conventional mechanical pretreatment ((Ziegler-Devin et al. 2021; T, Pielhop, et al. 2016; AT, Hoang., et al. 2023; NN, Idris., et al. 2025)

HTP selectively affects the different biomass components (Martín et al. 2022a). HTP's primary target is the amorphous hemicellulose fraction, which is effectively hydrolyzed and solubilized into the liquid hydrolysate, primarily as oligosaccharides and, under more severe conditions, as monomers like xylose (Fig. 2B) (Martín et al. 2022a; Zhang et al. 2018a). HTP does not typically remove a large fraction of lignin. Instead, it modifies its structure through depolymerization, primarily via the cleavage of β–O -4 ether linkages (Chen et al. 2018b). This fragmented lignin can then migrate and recondense onto the cellulose surface (Fig. 2B), a significant drawback that can reduce enzyme accessibility (Li et al. 2014; M, T., RP, C. JN, S. 2019; J, L., et al. 2019). The crystalline cellulose core is largely resistant to hydrolysis under typical HTP conditions (Martín et al. 2022a). However, the removal of the surrounding amorphous matrix (hemicellulose) can lead to the coalescence of cellulose microfibrils. This phenomenon can result in an increase in the measured cellulose crystallinity index (CrI) and crystal size, which can negatively impact digestibility if not offset by gains in porosity(Méndez-Durazno et al. 2025).

The principal benefit of HTP is the removal of the hemicellulose sheath, which dramatically increases the specific surface area (SSA) and pore volume of the cellulosic substrate(Chen et al. 2018a). This directly enhances the accessibility of cellulase enzymes. To contextualize the yield improvement over untreated biomass, Zhang et al*.* reported that hydrothermally pretreated corn stover exhibited a twofold increase in SSA, which directly resulted in a 138% enhancement in enzymatic digestibility compared to the native, untreated material (Zoghlami and Paes 2019; Zhang et al. 2018b). Despite its efficacy, HTP faces a major drawback: the generation of a complex cocktail of inhibitory compounds. This primarily occurs under the high-severity conditions, specifically elevated temperatures and prolonged retention times (Kakar et al. 2022), that are ironically required to maximize the initial release of sugars (Martín et al. 2022a). This creates a processing paradox where the treatment degrades the very sugars it aims to liberate.

These inhibitors fall into two distinct categories based on their chemical origin and the specific downstream step they disrupt. The first category comprises soluble degradation products that are potent inhibitors of downstream microbial fermentation. At high severity, pentose sugars, such as xylose (released from hemicellulose), dehydrate to form furfural. Simultaneously, hexose sugars like glucose (liberated from cellulose) degrade into 5-hydroxymethylfurfural (HMF; Fig. 2B) (Li et al. 2018), which can subsequently break down further into levulinic and formic acids (Martín et al. 2022a). The second category involves the most detrimental by-product of the HTP process: pseudo-lignin (Fig. 2B). Unlike the soluble furans and acids that inhibit microbes, pseudo-lignin directly inhibits the enzymatic saccharification stage. Under the acidic, high-temperature conditions of HTP, the aforementioned carbohydrate degradation products (especially HMF) can react and co-polymerize with depolymerized lignin fragments. This reaction forms a solid, lignin-like polymer, pseudo-lignin, which re-precipitates onto the surface of the cellulose (Martín et al. 2022a), physically blocking enzyme access and non-productively binding cellulases (Li et al. 2014).

To overcome the inhibitory effects of pretreatment-derived compounds on downstream bioconversion, detoxification strategies must be evaluated in the context of inhibitor spectrum, sugar retention, and process integration (Gyan et al. 2024; Lyu et al. 2025; KF, C., et al. 2025; Zhang et al. 2018c). Detoxification methods are commonly grouped into physicochemical and biological approaches (Gyan et al. 2024; Lyu et al. 2025). Physical and chemical methods, including overliming, activated carbon adsorption, solvent extraction, membrane separations, and related conditioning steps, can remove substantial amounts of soluble furanic and phenolic inhibitors from hydrolysates (Gyan et al. 2024; Lyu et al. 2025; KF, C., et al. 2025; Zhang et al. 2018c). However, these approaches add extra unit operations, increase reagent and utility demand, and may also remove or degrade a fraction of fermentable sugars (Gyan et al. 2024; Lyu et al. 2025; KF, C., et al. 2025; Zhang et al. 2018c). Biological detoxification, or bio-abatement, employs microorganisms or oxidative enzymes such as laccases to transform inhibitors, especially phenolic compounds, and can improve hydrolysate fermentability while often preserving sugars more effectively than harsher physicochemical conditioning. This advantage is offset by longer residence times, additional biocatalyst handling, and reactor-integration requirements (Ujor and Okonkwo 2022; MT, F.-S., et al. 2024; Haq et al. 2024). Consequently, alongside ex situ detoxification, growing attention is being directed toward in situ mitigation strategies, including the engineering of fermentation strains with improved hydrolysate tolerance and the use of additives such as nonionic surfactants or water-soluble lignins/lignosulfonates that reduce non-productive cellulase adsorption onto residual lignin-rich solids during saccharification (Tian et al. 2025; Yu et al. 2025; Yuan et al. 2021; Wang et al. 2023; Sánchez-Muñoz et al. 2022). Ultimately, the choice of detoxification strategy should be guided by rigorous techno-economic evaluation to balance inhibitor removal efficiency against added process complexity, sugar recovery, and cost.

This highlights a central challenge of HTP: the process operates under a severity crossover paradigm. Pretreatment effectiveness is often quantified by a severity factor (SF), which combines the effects of temperature and time (Méndez-Durazno et al. 2025). As SF increases, hemicellulose removal and enzyme accessibility improve, but only up to a point. A critical crossover is reached where the rate of inhibitor and pseudo-lignin formation (Li et al. 2014) begins to outpace the benefits of matrix disruption (Kellock et al. 2019; Batista et al. 2019). This creates a processing cliff where higher severity becomes counter-productive, resulting in yield loss due to sugar degradation and enzymatic inhibition (Martín et al. 2022a).

HTP optimization is therefore not a simple maximization problem, but a complex search for a sweet spot by tuning key operational parameters, primarily temperature, retention time, and the solid-to-liquid ratio. The objective is to maximize the positive pathways: specifically, the extensive solubilization and removal of the hemicellulose barrier, which directly increases the accessible surface area of cellulose for subsequent enzymatic hydrolysis. However, this must be carefully balanced against the negative, high-severity chemical degradation pathways. If the operational parameters are too severe, it not only degrades the liberated fermentable sugars but also generates two distinct classes of inhibitors: pseudo-lignin, which physically blocks and non-productively binds cellulases to negatively influence the hydrolysis step itself, and soluble degradation products like furfural, HMF, and organic acids, which severely impair the downstream microbial fermentation process.

### Rapid, selective, and volumetric heating via microwave-assisted pretreatment

Microwave-assisted pretreatment (MWP) represents a significant shift from conventional thermal methods, employing process intensification principles to enhance the deconstruction of biomass (Venegas-Vásconez et al. 2025). Instead of relying on slow conductive and convective heat transfer from an external source, MWP utilizes microwave radiation, typically at 2.45 GHz, to heat the material (Fig. 2C) (Goyal et al. 2022). The mechanism is based on dielectric heating, in which the radiation is directly absorbed by polar molecules within the biomass, primarily water, but also polar groups on the biopolymers, causing them to rapidly oscillate as they align with the changing electromagnetic field (Fernandes et al. 2023). This oscillation generates heat volumetrically and internally throughout the material (Fernandes et al. 2023).

This heating is also highly selective. The microwaves preferentially heat the more polar components, creating localized hotspots within the biomass structure (Fig. 2C) (Venegas-Vásconez et al. 2025). This leads to rapid internal pressure build-up, resulting in micro-explosion effects that physically disrupt the biomass structure (Fig. 2C) (Venegas-Vásconez et al. 2025). Furthermore, some studies have proposed the existence of non-thermal effects, where the electromagnetic field itself may contribute to bond cleavage by lowering activation energies, though this remains a topic of investigation (Nomanbhay, et al. 2013).

The unique heating mechanism of MWP facilitates distinct structural and chemical changes, as the rapid, selective heating is highly effective in promoting hemicellulose depolymerization and, significantly, weakening lignin-carbohydrate linkages (LCCs) (Venegas-Vásconez et al. 2025). MWP has also been shown to be effective in reducing cellulose crystallinity, thereby further enhancing enzyme accessibility to the carbohydrate fraction, often through delignification and structural disruption (Venegas-Vásconez et al. 2025; Mikulski et al. 2022; Chen and Wan 2018). However, the relationship between reduced crystallinity and increased sugar yield is complex, and some studies have noted increased sugar yields even with slightly higher crystallinity, suggesting that the degree of delignification and overall accessibility play a dominant role in those cases (Zeng et al. 2022).

MWP can substantially enhance enzymatic saccharification within minutes, compared with the hours often required for conventional LHW-HTP, although its reaction times are more comparable to those of steam explosion (Fernandes et al. 2023). Multiple studies have reported high reducing sugar yields (Zeng et al. 2022) For example, Nomanbhay et al. reported a remarkable 5.8-fold enhancement in sugar yield from microwave-pretreated samples over native, untreated samples (Nomanbhay, et al.^ 2013^). The rapid process limits the time available for sugar degradation reactions; consequently, microwave-assisted hydrothermal (MW-HT) or microwave-acid processes can often achieve high sugar yields with lower concentrations of inhibitors such as furfural and HMF than conventional HTP (Ethaib et al. 2016).

**Fig. 3:**
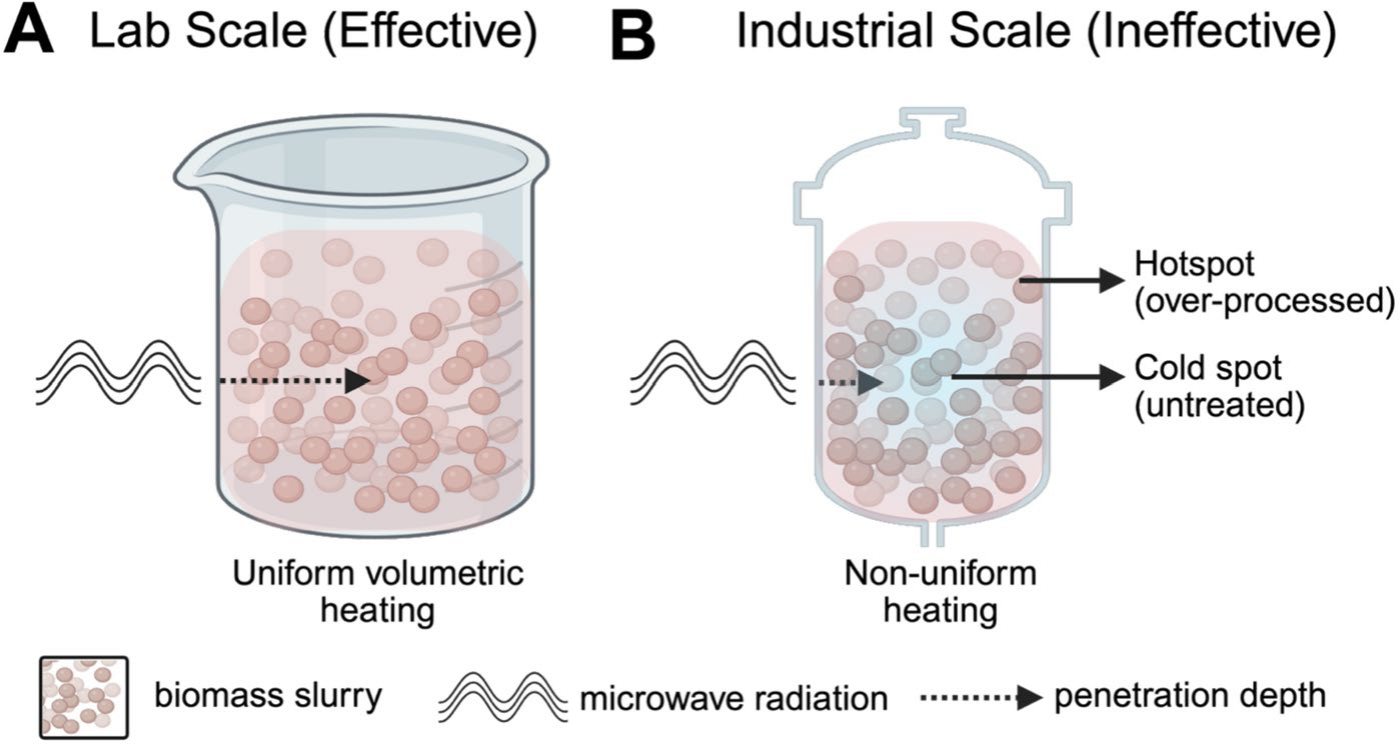
The industrial scalability challenge of microwave pretreatment (MWP). This figure illustrates the fundamental engineering bottleneck that limits the commercial scale-up of MWP, derived from the physics of microwave matter interaction. Microwave energy absorption and heat generation are governed by a material's dielectric properties and its loss factor (Venegas-Vásconez et al. 2025). Penetration depth, indicated by dashed arrow, is the distance microwaves effectively penetrate and is inversely proportional to the loss factor of the material being heated (Sun et al. 2016). **A** In small, laboratory-scale reactors, the sample volume is small enough that the microwave penetration depth easily spans the entire reaction volume (John Hokanson et al. 2011; Antunes et al. 2019). This results in uniform volumetric heating and reproducible, efficient pretreatment (indicated by red area). **B** Conversely, when scaled to large industrial volumes, the finite penetration depth, typically only a few centimeters at 2.45 GHz, fails to reach the reactor center (Syed et al. 2023; John Hokanson et al. 2011). This leads to a non-uniform distribution of the electromagnetic field, resulting in localized areas near the microwave source that receive intense, runaway heating hotspots (Syed et al. 2023; Hoz, et al. 2011) (red area), while the large central mass of biomass remains untreated, with cold spots (Syed et al. 2023; John Hokanson et al. 2011) (blue area). This inconsistency necessitates complex and costly reactor designs, such as continuous flow systems or thin-layer applicators, to ensure uniform treatment before commercial deployment (Siddique et al. 2022; Mitani 2018). Images were created using BioRender

The primary Achilles' heel of MWP is the significant challenge of scaling the technology from the lab bench to a commercial, pilot-scale process (Fig. 3A, B) (Siddique 2021). This challenge is rooted in the fundamental physics of microwave-matter interactions, which are highly dependent on the material's dielectric properties and penetration depth. Specifically, at an industrial scale, the finite microwave energy penetration depth, which is typically only a few centimeters at 2.45 GHz (Goyal et al. 2022), makes it physically impossible to uniformly heat the contents of large-diameter reactors (Fig. 3B) (Syed et al. 2023). Furthermore, non-uniform electromagnetic field distribution creates hotspots, which are localized areas of high temperature, and cold spots within large cavities (Syed et al. 2023), leading to inconsistent pretreatment, potential thermal damage, and safety hazards (Fig. 3B) (Hoz, et al. 2011). Overcoming these issues necessitates moving away from batch processing entirely and designing complex, expensive continuous-flow systems, such as conveyor belts (Al-Qahtani and Al-Qahtani 2023) or specialized, pressurized reactors made of microwave-transparent materials, like quartz (Cabrera et al. 2025). This adds immense capital expenditure (CapEx) and complexity, posing a severe scale-up hurdle(Siddique 2021) that currently limits MWP's commercial viability compared to mature technologies like steam explosion.

This presents a fundamental engineering paradox: the very physics that make MWP uniquely effective at the lab scale are the same physics that cause it to fail at an industrial scale. The process relies on the interaction of microwaves with the material's dielectric properties, specifically its loss factor, which governs the conversion of electromagnetic energy into heat (Venegas-Vásconez et al. 2025). A high loss factor is desirable for rapid heating. However, a high loss factor is also inversely related to penetration depth (Sun et al. 2016). Therefore, the mechanism of action is simultaneously the mechanism of failure at scale. This realization dictates that the future of MWP is not in optimizing chemistry (which is well understood) but in novel reactor engineering, such as continuous-flow and thin-film systems (Al-Qahtani and Al-Qahtani 2023), that can effectively manipulate the physics of penetration depth.

### Nanoscale disruption of crystalline cellulose using mechanochemical ball milling pretreatment

Ball milling (BM) is a physical, top-down pretreatment that operates on mechanochemical principles (Ramirez-Cabrera and Ramirez-Cabrera 2024). It is a high-energy milling process where biomass is loaded into a rotating or vibrating jar, along with milling media, such as steel or ceramic balls. The process involves high-energy collisions between the milling balls and the biomass, which imparts intense mechanical forces, including shear, impact, and friction, that physically deconstruct the plant cell wall at a micro- and nanoscale (Sitotaw et al. 2021) (Fig. 2D).

Unlike chemically selective methods like HTP, BM is a brute-force approach that nonselectively affects all components of the biomass by reducing particle size and dramatically increasing the SSA (Sitotaw YW et al 2021). Wu et al*.* found that BM increased the SSA of cellulose by approximately two-fold, making it more accessible and reactive (Zoghlami and Paes 2019; Venegas-Vásconez et al. 2025; Wu et al. 2018). However, the most important and distinctive effect of BM is the destruction of the crystalline structure of cellulose. The intense mechanical energy overcomes the extensive hydrogen bonding that maintains the crystalline lattice, resulting in a significant reduction in both cellulose crystallinity and degree of polymerization (Gu et al. 2018; Mattonai et al. 2018). The disruption of cellulose crystallinity is central to BM pretreatment because it dramatically accelerates hydrolysis kinetics.

With prolonged milling, the recalcitrant cellulose I allomorph can be converted into a highly disordered, amorphous state (Gu et al. 2018; Mattonai et al. 2018). Interestingly, in aqueous media, this amorphous cellulose may subsequently recrystallize into cellulose II, an allomorph that remains more accessible to enzymes and water, thereby enhancing subsequent hydrolysis. The destruction of cellulose crystallinity is the single most important factor for BMs success in pretreatment. The kinetics of enzymatic hydrolysis are dramatically enhanced because cellulases no longer need to expend energy deconstructing a highly ordered crystalline lattice; they can directly attack the now amorphized chains (Wu et al. 2018; Yu and Wu 2011; Mais, et al. 2002). Consequently, while untreated lignocellulosic biomass typically exhibits very low baseline saccharification yields due to crystallinity, BM provides a massive multi-fold enhancement, resulting in nearly theoretical glucose yields (> 90% conversion) from the pretreated substrate(Yu and Wu 2011) (Fig. 2D).

The primary drawback of BM is its prohibitively high energy consumption (Ramirez-Cabrera and Ramirez-Cabrera 2024; Zhao, et al. 2005; Blasi et al. 2022). It is an exceptionally energy-intensive process, which makes its techno-economic feasibility as a standalone pretreatment highly questionable (Sitotaw et al. 2021). A techno-economic analysis based on a three-stage milling process reported a consumption of 2.05 kJ/g for the final media milling stage alone (Brandt et al. 2018), while other studies have reported figures as high as 10.08 kJ/g for ball milling (Brandt et al. 2018). This high operational cost is a major barrier to industrial adoption (Blasi et al. 2022). A major advantage, however, is that, as a purely physical process run without chemical additives, BM generates little to no chemical inhibitors such as furfural and HMF (Baruah, et al. 2018; Yang et al. 2022). This significantly simplifies downstream fermentation, as it entirely avoids the need for the complex and costly detoxification steps (discussed earlier in the context of HTP).

This positions BM's true value not as a standalone pretreatment, but as a potent process intensifier. Unlike HTP modalities such as steam explosion, which primarily separate components by solubilizing hemicellulose and often result in an increase in the relative crystallinity index of the remaining solid due to the loss of amorphous material, BM fundamentally changes the cellulose substrate itself. It actively converts the material from a highly ordered, recalcitrant crystalline lattice into a highly reactive amorphous state (Yu and Wu 2011). This brute force approach, while energetically inefficient on its own (Ramirez-Cabrera and Ramirez-Cabrera 2024; Brandt et al. 2018), can be strategically deployed to trade high electrical energy input for massive savings in thermal and chemical energy downstream. By amorphizing the cellulose, BM enables subsequent chemical or thermal steps to be performed under much milder conditions (Fig. 2D). For example, a mild HTP step, which would be ineffective on its own, can achieve near-complete hydrolysis on ball-milled biomass without generating inhibitors (Zakaria et al. 2014; Gladysheva 2025). Therefore, the role of BM in a modern biorefinery is not to replace other methods, but to enable them to work under greener, cheaper, and more efficient conditions.

### Critical comparative analysis and synergistic integration

An effective pretreatment must balance four key metrics: saccharification efficacy, inhibitor formation, energy consumption, and scalability. The three technologies reviewed here illustrate a clear set of mechanistic and techno-economic trade-offs.

1. Saccharification efficacy is the primary performance metric for any pretreatment strategy. HTP improves enzymatic digestibility primarily by solubilizing hemicellulose and disrupting lignin–carbohydrate linkages, thereby increasing cellulose accessibility. MWP can achieve similar improvements in much shorter residence times due to rapid volumetric heating. BM is particularly effective at enhancing saccharification because it reduces particle size, increases surface area, and markedly decreases cellulose crystallinity. However, the magnitude of improvement depends strongly on biomass type and process severity, which is why hybrid systems often outperform standalone pretreatments.
2. Inhibitor formation is a key differentiator. HTP carries the highest risk, generating a complex cocktail of furfurals, organic acids, and, most problematically, pseudo-lignin, which often necessitates a separate detoxification step (Woźniak et al. 2025; Martín et al. 2022a; Kellock et al. 2019; Wan et al. 2019). BM is the cleanest of the three, producing negligible chemical degradation products (Baruah, et al. 2018; Yang et al. 2022). MWP falls in the middle; its rapid processing generally mitigates inhibitor formation compared to conventional HTP operating at the same severity, although it does not eliminate inhibitor formation entirely (Chen and Wan 2018; Ethaib et al. 2016; Shukla et al. 2023).
3. Energy consumption and economics present the most complex trade-off. BM is widely cited as the most energy-intensive process, requiring a massive electrical input for mechanical grinding(Ramirez-Cabrera and Ramirez-Cabrera 2024; Gu et al. 2018; Brandt et al. 2018). Conversely, the SE modality of HTP can be more energy-efficient for size reduction than mechanical milling. Furthermore, comparisons among thermal methods also suggest that MWP can be more energy-efficient than conventional, conductively heated HTP systems (whether LHW or SE), as demonstrated by a study where it achieved 40.1 kJ/g compared to 70.85 kJ/g. This efficiency is attributed to its volumetric heating (Rouabhia et al. 2025). However, a critical distinction must be made between the quantity and quality of energy used. MWP relies on high-grade, expensive electricity, whereas HTP can potentially be integrated with a biorefinery's combined heat and power unit, enabling it to operate on low-grade waste heat (Liu and Bao 2017). This heat integration capability could make HTP far more economically viable than MWP in a full-scale facility (Saritpongteeraka et al. 2020).
4. HTP, particularly steam explosion, is the most mature technology and has been demonstrated at industrial and pilot scales (Martín et al. 2022a; Eom et al. 2019; Putra 2018). BM is also a scalable technology used in mining and pharmaceuticals, but its prohibitive energy cost, not a technical hurdle, remains the barrier to its use in a biorefinery (Gu et al. 2018; Blasi et al. 2022). MWP is the least mature. It faces fundamental scientific and engineering hurdles, such as penetration depth, hotspots, and reactor design, that make scale-up a nonlinear, complex, and expensive challenge (Siddique 2021; Syed et al. 2023; John Hokanson et al. 2011; Siddique et al. 2022).
5. The limitations inherent in each individual pretreatment method, namely HTP's associated inhibitor formation, MWP's severe scalability challenges, and BM's high energy consumption, are driving the field toward hybrid and synergistic pretreatment strategies that intentionally leverage the distinct strengths of each technology to offset their weaknesses (Kumar et al. 2009; Kim and J. H. C., Bong-Yong Jeong 2013). The combination of BM followed by LHW-HTP is particularly promising because BM first reduces cellulose crystallinity (Gu et al. 2018; Mattonai et al. 2018; Gladysheva 2025), allowing the subsequent LHW step to operate under milder conditions, lower temperature, and shorter reaction time. This mild LHW effectively removes hemicellulose from the newly accessible matrix without the high severity required to produce degradation inhibitors. This synergy is clearly demonstrated by a combined alkaline hydrothermal treatment (which incorporates a mild base, such as sodium hydroxide, into the aqueous system to actively promote delignification alongside hemicellulose solubilization) and BM, which together achieve a near-theoretical glucose yield of 97.3% (Yang et al. 2022; Zakaria et al. 2014). Similarly, combining microwave and hydrothermal treatment functions as a best-of-both-worlds thermal strategy (Gao et al. 2021), by leveraging the rapid, volumetric, and selective heating of MWP (Venegas-Vásconez et al. 2025; Chen and Wan 2018; Brandt et al. 2018; Ren et al. 2020) to accomplish the core chemical objectives of HTP within a shorter timeframe, thereby minimizing the duration available for undesirable sugar degradation reactions and subsequent inhibitor formation (Gao et al. 2021).

Table 1 presents a comparative analysis of hydrothermal, microwave, and ball milling pretreatments. This comparison evaluates key metrics and mechanisms, including effects on lignocellulose components, inhibitor formation, energy consumption, scalability, and operational drawbacks.

**Table 1:** Comparative feature analysis of hydrothermal, microwave, and ball milling pretreatments

Feature	Hydrothermal pretreatment (HTP)	Microwave assisted pretreatment (MWP)	Ball milling (BM)
Primary mechanism	Hemicellulose hydrolysis autohydrolysis(Martín et al. 2022a; Ma et al. 2025)	Dielectric heating; selective cell wall disruption(Venegas-Vásconez et al. 2025; Fernandes et al. 2023)	Mechanochemical; crystallinity destruction (Sitotaw et al. 2021; Pérez-Merchán et al. 2022)
Effect on cellulose	Minor fragmentation; crystallinity may increase(Martín et al. 2022a; Méndez-Durazno et al. 2025)	Reduced crystallinity; structural disruption(Venegas-Vásconez et al. 2025; Ren et al. 2020)	Severe crystallinity reduction amorphization/Cellulose II (Yu and Wu 2011; Lan et al. 2022)
Effect on hemicellulose	High solubilization and hydrolysis(Martín et al. 2022a; Méndez-Durazno et al. 2025)	Depolymerization(Venegas-Vásconez et al. 2025)	Partial disruption; aids in later removal (Qu et al. 2017)
Effect on lignin	Modification; relocation; recondensation (Li et al. 2014; Chen et al. 2018b)	Partial removal; weakened LCCs(Venegas-Vásconez et al. 2025; Ethaib 2024)	Depolymerization; structural disruption (Sitotaw et al. 2021)
Key inhibitors formed	High Furfural, HMF, pseudo lignin(Martín et al. 2022a; Świątek et al. 2020)	Moderate shorter time mitigates(Ethaib et al. 2016; Shukla et al. 2023)	Negligible(Baruah, et al. 2018)
Energy consumption	High Thermal(Woźniak et al. 2025); can use waste heat(Saritpongteeraka et al. 2020)	Moderate Electricity; efficient but high-grade energy(Rouabhia et al. 2025; Saritpongteeraka et al. 2020)	Very High Mechanical Electricity (Ramirez-Cabrera and Ramirez-Cabrera 2024; Brandt et al. 2018)
Scalability status	Industrially demonstrated(Martín et al. 2022a; Putra 2018)	Challenging penetration depth, hotspots(Siddique 2021; John Hokanson et al. 2011; Siddique et al. 2022)	Challenging energy cost, throughput (Blasi et al. 2022)
Primary advantage	Effective hemicellulose removal; mature tech(Martín et al. 2022a)	Rapid heating; high selectivity; lower inhibitors than HTP(Fernandes et al. 2023; Gao et al. 2021; Kumar and Sharma 2017)	Drastic increase in enzyme kinetics; no inhibitors (Yu and Wu 2011; Mais, et al. 2002; Baruah, et al. 2018)
Primary drawback	Inhibitor formation; pseudo lignin(Martín et al. 2022a)	Scalability; high CapEx reactor(Siddique 2021; Syed et al. 2023; John Hokanson et al. 2011)	Prohibitive energy consumption (Ramirez-Cabrera and Ramirez-Cabrera 2024; Zhao, et al. 2005)

Table 2 provides a concise side-by-side comparison of hydrothermal pretreatment (HTP), microwave-assisted pretreatment (MWP), and ball milling (BM) in terms of representative reaction conditions, relative cost, inhibitor profile, effects on lignin and hemicellulose, and associated energy demand. This comparison complements the conceptual summary in Table 1 by highlighting the main operational and techno-economic trade-offs among the three pretreatment routes. As shown, HTP is most effective for hemicellulose solubilization but carries a higher risk of inhibitor formation, MWP offers faster processing with intermediate trade-offs, and BM is distinguished by strong physical disruption with minimal inhibitor generation but high electrical energy demand.

**Table 2:** Comparative operational, compositional, and techno-economic analysis of hydrothermal, microwave, and ball milling pretreatments

Parameter	Hydrothermal pretreatment (HTP)	Microwave-assisted pretreatment (MWP)	Ball milling (BM)
Typical reaction conditions	Water-based; typically ~ 160–240 °C under autogenous pressure; residence time from minutes to hours depending on severity and mode (LHW vs SE)	Microwave heating, often with water or dilute acid/alkali; typically ~ 120–220 °C equivalent bulk temperature; residence time usually minutes	Ambient to moderately elevated temperature; mechanical milling for minutes to hours depending on mill type, speed, and biomass loading
Relative cost associated	Moderate; attractive because water is the main medium, but reactor pressure requirements and downstream conditioning add cost	Moderate to high; short residence time can lower processing time, but microwave equipment, electricity demand, and scale-up constraints increase cost uncertainty	High as a standalone step because of substantial electrical energy demand and wear of milling equipment
Inhibitor profile	Highest risk among the three; can generate furfural, HMF, organic acids, phenolics, and pseudo-lignin at higher severity	Usually lower inhibitor formation than conventional HTP at comparable sugar release because of shorter heating times, but furfural/HMF can still form under severe conditions	Minimal chemical inhibitor formation because the process is physical rather than thermochemical
Effect on lignin / lignin removal	Limited bulk lignin removal; mainly lignin redistribution, depolymerization/recondensation, and possible pseudo-lignin formation	Usually limited bulk lignin removal unless chemicals are added; can alter lignin structure and improve accessibility	No selective lignin removal; mainly physical disruption and increased surface exposure
Effect on hemicellulose / hemicellulose removal (%)	Strongest hemicellulose solubilization among the three; removal is severity-dependent and can range from partial to substantial	Moderate to high when combined with water, acid, or alkali; often similar in objective to HTP but achieved more rapidly	Very limited direct hemicellulose solubilization/removal; effect is mainly physical rather than chemical
Energy associated	Mainly thermal energy; can potentially integrate with waste heat or steam systems	Mainly high-grade electrical energy; may be efficient in rapid heating but depends strongly on reactor design and scale	Highest electrical energy demand among the three; major barrier to standalone commercialization
Main mechanistic effect	Autohydrolysis of hemicellulose and disruption of lignin–carbohydrate linkages	Rapid volumetric dielectric heating that accelerates hydrothermal/chemical reactions	Reduction in particle size, increase in surface area, and strong decrease in cellulose crystallinity
Main strength	Mature, widely studied, effective for opening biomass structure and solubilizing hemicellulose	Fast processing and potentially lower inhibitor formation than prolonged thermal treatment	Excellent cellulose decrystallization and strong enhancement of enzymatic digestibility without generating inhibitors
Main limitation	Inhibitor formation and lignin recondensation at higher severity	Scale-up limitations, penetration depth, hotspot formation, and electricity dependence	Prohibitively high energy consumption if used alone

### Valorization strategies in a circular bioeconomy

The economic viability of a lignocellulosic biorefinery hinges on the complete valorization of all biomass components, not just cellulose (Ubando et al. 2020; Tanis et al. 2023; Devi., et al. 2022). The pretreatment step is not just about preparing cellulose; it is the key fractionation step that dictates the value of the hemicellulose and lignin co-product streams.

**Fig. 4:**
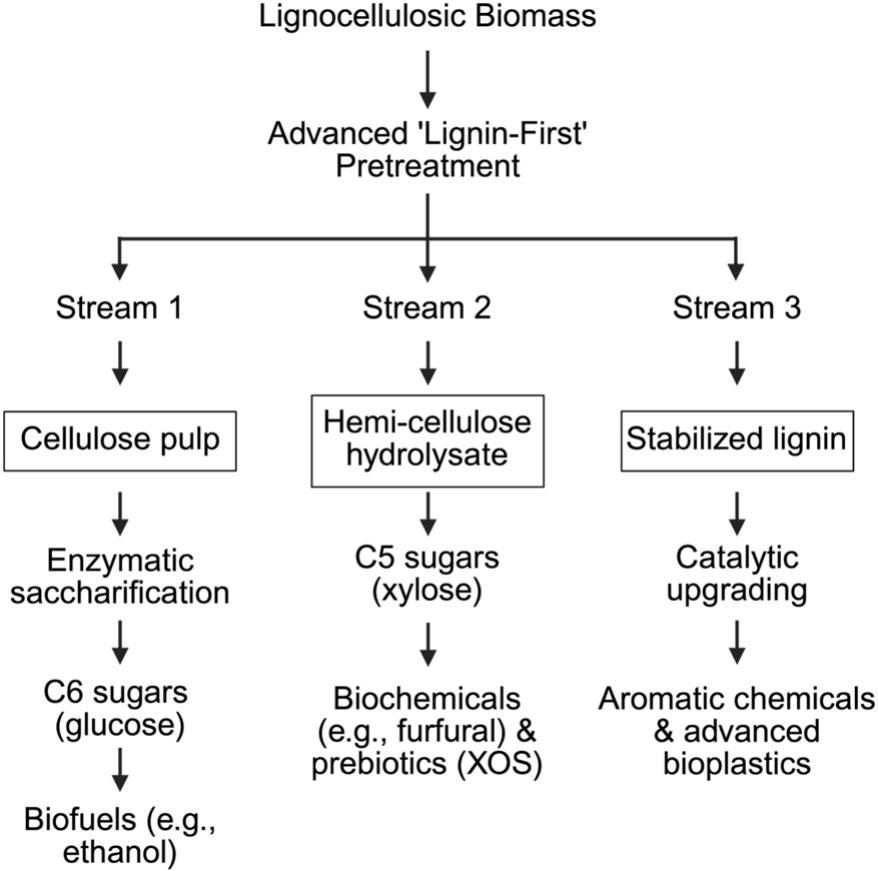
Integrated schematic of a lignin-first biorefinery within a circular bioeconomy framework. This flowchart illustrates the idealized objective of modern lignocellulosic biorefining, in which the pretreatment stage functions not only as a deconstruction step but also as the central fractionation step that determines the downstream value of all three major biomass components (Tanis et al. 2023; A, D., et al. 2022). In this framework, advanced lignin-first pretreatment is designed to suppress lignin recondensation reactions and generate a clean, reactive, and stabilized lignin stream, thereby minimizing the formation of intractable pseudo-lignin (Brienza, et al. forthcoming). The resulting fractionation produces three parallel product streams. First, the cellulose-rich pulp is directed to enzymatic saccharification to generate C6 sugars, primarily glucose, which serve as key intermediates for second-generation biofuels such as ethanol (A, D., et al. 2022; Atitallah et al. 2022). Second, the hemicellulose-derived hydrolysate, containing C5 sugars and soluble oligomers such as xylose, can be upgraded into high-value platform biochemicals or prebiotic products such as xylooligosaccharides (XOS) (Pinales-Márquez et al. 2021). Third, the stabilized, non-condensed lignin stream is catalytically upgraded or chemically modified into aromatic chemicals and advanced biomaterials, thereby maximizing overall revenue and accounting for over 40% of the original energy content (Pinales-Márquez et al. 2021; Brienza, et al. forthcoming; Ullah et al. 2022)

The liquid hydrolysate from HTP or MWP is not a waste stream. It is a rich source of C5 and C6 sugars that can be fermented to biofuels (Pinales-Márquez et al. 2021) (Fig. 4), and under controlled conditions, can also be routed to co-produce fermentable sugars and hemicellulose-derived oligomers, such as xylooligosaccharides (XOS) (Su et al. 2021; Zhang et al. 2022; Fang et al. 2022; Zhu et al. 2023). Under carefully controlled HTP conditions, hemicellulose can be recovered as oligomers, specifically XOS, which are high-value prebiotics for the food and pharmaceutical industries (Fig. 4) (Rouabhia et al. 2025; Pinales-Márquez et al. 2021; Su et al. 2021; Zhang et al. 2022; Fang et al. 2022; Zhu et al. 2023).

Additionally, lignin is the most critical co-product for biorefinery economics, potentially accounting for over 40% of the energy content of the biomass (A, D., et al. 2022). The choice of pretreatment fundamentally dictates the chemical structure, purity, and subsequent valorization potential of the resulting lignin fraction (Tanis et al. 2023; Mankar et al. 2022; Xu et al. 2020; Brienza, et al. forthcoming). HTP, for example, can produce depolymerized lignin with cleaved β-O-4 bonds, which may be suitable for catalytic upgrading (Chen et al. 2018b; Syed et al. 2023). However, it also carries a high risk of recondensation into intractable pseudo-lignin, which reduces lignin value.

This challenge has given rise to the lignin-first biorefinery concept (Fig. 4) (Filippo Brienza et al. 2024; Chen L et al. 2021) This is an advanced pretreatment strategy, often involving catalysis, designed to simultaneously depolymerize and stabilize reactive lignin intermediates, actively preventing recondensation (Cabrera et al. 2025; Brienza, et al. forthcoming; Ethaib et al. 2020a). This approach fractionates the biomass into three clean, high-value streams: a highly digestible cellulose pulp, a hemicellulose-rich liquid stream, and a reactive, depolymerized lignin oil suitable for conversion into aromatic chemicals or advanced biomaterials (Brienza, et al. forthcoming; Chen et al. 2021). While lignin-first approaches such as reductive catalytic fractionation (RCF) offer a promising route for maximizing lignin valorization while preserving carbohydrate pulps, their scale-up introduces important environmental and process-integration trade-offs (Bartling et al. 2021; Daelemans et al. 2025; Arts et al. 2023). RCF commonly relies on a catalyst, a hydrogen source or hydrogen-donor strategy, and substantial solvent inventories, making solvent handling and recovery central to both process economics and environmental performance (Bartling et al. 2021; Daelemans et al. 2025; Arts et al. 2023). In many recent techno-economic and life-cycle studies, downstream solvent/water separation and solvent recycle are identified as major contributors to process heat demand, operating cost, and global warming potential (GWP) (Bartling et al. 2021; Arts et al. 2023). Accordingly, the sustainability of RCF should be viewed as highly sensitive to solvent-management strategy: high solvent-recovery or liquor-recycle rates can substantially improve cost and GWP outcomes, but the required recovery level is process-specific rather than defined by a universal threshold (Bartling et al. 2021; Daelemans et al. 2025; Arts et al. 2023). Catalyst selection and recyclability remain additional considerations, particularly in systems using expensive or scarce metals (Daelemans et al. 2025; Bugli et al. 2024).

This illustrates a paradigm shift in the philosophy of pretreatment. Pretreatment is not merely a means of unlocking cellulose; it is the central fractionation step that determines the fate and value of each biomass component. Methods like high-severity HTP, which damage co-product streams by, for example, creating pseudo-lignin, are leaky buckets in a circular bioeconomy. In contrast, lignin-first approaches (Brienza, et al. forthcoming) or well-designed hybrid methods that produce clean, separable fractions (Zakaria et al. 2014), represent a more holistic and economically sustainable model for the biorefinery of the future.

### Integrated process analysis: chemistry, biology, and economics

An integrated evaluation framework is essential for translating lignocellulosic pretreatment from laboratory-scale success to industrial implementation. In practice, pretreatment performance cannot be assessed solely by chemical fractionation efficiency, because its true value depends on how it interacts with downstream biological conversion, inhibitor management, product valorization, and overall process economics. This systems-level perspective is particularly important for comparing HTP, MWP, and BM, as each technology introduces distinct trade-offs in structural disruption, inhibitor formation, energy demand, and scalability. Accordingly, this section examines pretreatment within an integrated process framework that connects chemical deconstruction, biological compatibility, and techno-economic viability.

Beyond physicochemical approaches, specific chemical and biological strategies are employed to address the full spectrum of lignocellulose recalcitrance. Organosolv pretreatment, for instance, represents a pivotal chemical pathway facilitating the simultaneous recovery of high-purity lignin, cellulose, and hemicellulose sugars (Mankar et al. 2022; Nair et al. 2023; Vaidya et al. 2022). Validated at the pilot scale (Mankar et al. 2022; Tofani et al. 2024; Smit et al. 2022), the inclusion of organosolv is critical for reinforcing lignin-first valorization strategies. Similarly, alkaline pretreatment, such as sodium hydroxide, remains highly relevant due to its selectivity; it addresses recalcitrance primarily through delignification rather than solely relying on matrix disruption (Qu et al. 2017; N, K., EM, B., S, M., D, M. M, L. 2019; W et al. 2018). From a sustainability and biotechnological perspective, biological pretreatment utilizing white-rot fungi, such as *Phanerochaete chrysosporium *(Tanis et al. 2023; Tamo et al. 2025), offers a lower energy and chemical footprint (Rouabhia et al. 2025; C et al. 2017; W et al. 2020; Zhao et al. 2022; W and M 2011; Vasco-Correa et al. 2019). Although biological pretreatment is traditionally slower, advances in synthetic biology are beginning to reduce long residence times, supporting its continued relevance for sustainable biomass valorization (Zhao et al. 2022).

Despite the efficacy of HTP, its utility is often constrained by the formation of inhibitors (Li et al. 2014; Ruiz et al. 2020). Understanding the molecular mechanisms governing enzyme loss and inhibition is therefore crucial. A primary bottleneck is the non-productive adsorption of expensive cellulase enzymes onto the hydrophobic surfaces of residual lignin and pseudo-lignin, which directly reduces the enzyme concentration available for cellulose hydrolysis (Martín et al. 2022a; Lan et al. 2022). Furthermore, soluble inhibitors, including furan aldehydes such as HMF and furfural, and lignin-derived phenolics like vanillin, induce enzyme deactivation through chemical interactions and molecular binding (Qin et al. 2016; Ximenes et al. 2011; Xing 2016). Mitigation strategies are essential to overcome these barriers, including the use of inexpensive surfactants, such as Tween (Hou et al. 2022) or lignosulfonates, to preferentially block hydrophobic lignin sites (Xing 2016; Qing et al. 2010), or the genetic engineering of fermentation strains designed to tolerate elevated inhibitor concentrations (Rouabhia et al. 2025; Chauhan et al. 2024).

To transition from qualitative assessments to robust process viability, quantitative metrics are required. HTP (Putra 2018) and MWP (Saritpongteeraka et al. 2020) exhibit different energy-input, equipment, and scale-up profiles, and these differences should be assessed using both life cycle assessment (LCA) and techno-economic metrics (Świątek et al. 2020; Wang et al. 2020). Relevant benchmarks include water consumption, greenhouse-gas emissions (Woźniak et al. 2025), and the minimum sugar selling price (MSSP) of monomeric sugars (Wang et al. 2020; Brodeur et al. 2011).

Supporting these economic metrics requires advanced analytical infrastructure. The characterization of lignin fragments for valorization relies on techniques such as two-dimensional heteronuclear single quantum coherence nuclear magnetic resonance (2D-HSQC NMR) to quantify β-O-4 ether linkages (Kakar et al. 2022; Mankar et al. 2022; Liu et al. 2021a). Finally, the realization of smart biorefineries depends on the integration of real-time sensors, such as near-infrared (NIR) spectroscopy coupled with machine learning algorithms (Blasi et al. 2022; Kumar et al. 2009; Makaveckas et al. 2025), to monitor reactor conditions and dynamically adjust severity factors.

From a commercial perspective, benchmarking biomass valorization routes against incumbent fossil-derived fuels and chemicals remains essential, yet large-scale deployment of lignocellulosic biorefineries still faces substantial cost and scale-up barriers (Patel et al. 2025; Karimi et al. 2025; Sulis et al. 2025). At present, many second-generation biofuel pathways continue to struggle to achieve unsubsidized price parity with petroleum-derived fuels, and for sugar-platform biorefineries, the MSSP is a key economic metric because it strongly influences downstream fuel and chemical costs (Patel et al. 2025; Karimi et al. 2025; Brown et al. 2024; Baral et al. 2025). Economic competitiveness is therefore more likely in integrated multiproduct biorefineries, where valorization of hemicellulose and lignin into higher-value co-products, such as XOS, lignin-derived aromatics, or other specialty chemicals, can materially offset pretreatment and overall biorefinery costs, although the magnitude of this benefit remains pathway-dependent (Baral et al. 2025; Z et al. 2022; Liu et al. 2021b; Lan et al. 2021; Luo, et al. 2023). Commercial performance may be further improved by AI/ML-enabled monitoring and optimization that reduce energy and resource use, while stronger carbon-pricing policies can improve the relative economics of low-carbon routes by increasing the cost of fossil-intensive alternatives; however, both should be framed as enabling conditions rather than strict prerequisites (Butean et al. 2025; Döbbeling-Hildebrandt et al. 2024).

To strengthen the environmental comparison, life cycle assessment should include not only economic metrics such as MSSP but also comparative indicators such as global warming potential (GWP), cumulative energy demand, fossil resource scarcity, water consumption, and process-stage hotspots (Patel et al. 2025; Zhao et al. 2023). For example, a recent study on biosugar production reported GHG emissions as low as 0.03 kg CO_2_-eq per kg biosugar for corn stover deacetylation plus dilute-acid pretreatment (Balchandani et al. 2025), while another comparative TEA/LCA showed that MWP reduced cumulative energy demand by 5.82%, global warming impact by 4.85%, and fossil resource scarcity by 5.69% relative to conventional heating, although raw material extraction and solvent-recovery stages remained the dominant environmental hotspots (Wang et al. 2024b).

### Techno-economic assessment and machine learning applications

To transition pretreatment technologies from laboratory demonstration to industrially relevant deployment, eTEA and predictive digital tools are increasingly required to screen scale-up potential and identify major cost drivers (Patel et al. 2025; Wang et al. 2024b; Poveda-Giraldo et al. 2023). A key trade-off is that HTP and MWP exhibit different energy-input, equipment, and scale-up profiles; accordingly, comparative assessment should benchmark these routes using standardized indicators such as the MSSP of lignocellulosic sugars, together with capital and operating costs, rather than assuming a universal economic advantage for either route (Patel et al. 2025; WH, C., et al. 2022; Baral et al. 2025; Wang et al. 2024b; Martín et al. 2022b; Ou et al. 2021).

Furthermore, because lignocellulosic feedstocks vary substantially in composition and recalcitrance, fixed pretreatment conditions are rarely optimal across biomass types (WH, C., et al. 2022; Martín et al. 2022b; Ahmed et al. 2025). The field is therefore moving toward data-driven biorefineries in which rapid feedstock characterization tools, including NIR spectroscopy, are coupled with ML models for composition prediction, soft sensing, and process optimization (Ahmed et al. 2025; Nisar et al. 2025; Huang et al. 2025). Such models are being developed to relate feedstock properties and pretreatment operating variables to deconstruction performance and sugar yields, thereby supporting adaptive tuning of pretreatment severity-related conditions to improve carbohydrate release while reducing overprocessing; however, fully closed-loop industrial implementation should still be described as an emerging capability rather than established routine practice (Nisar et al. 2025; Huang et al. 2025; Xie and Fan 2025; Vuong et al. 2025).

### Future directions

The insights from this review highlight several critical areas for future research and development to accelerate the industrial deployment of these pretreatment technologies. The future does not lie in finding a single best pretreatment, but in designing intensified, integrated processes that are feedstock-agnostic and product-focused (Yu and Wu 2011; Sadula et al. 2017; Tang et al. 2024; Chen et al. 2024). Currently, the state of the art for hybrid methods (e.g., BM-HTP and MW-HT-alkali) demonstrates near-theoretical yields almost exclusively at the laboratory bench scale. Therefore, future development must focus on translating these synergistic batch processes into continuous, pilot-scale operations to truly minimize trade-offs (Venegas-Vásconez et al. 2025; Kim and J. H. C., Bong-Yong Jeong 2013; Qu et al. 2017).

Furthermore, while techno-economic assessments (TEAs) are common, they are often conducted retrospectively on relatively mature processes. Going forward, the field must prioritize early-stage TEA (eTEA) so that development of new hybrid methods directly targets reductions in the minimum selling price of fermentable sugars. Regarding downstream integration, while simultaneous saccharification and fermentation (SSF) is maturing, consolidated bioprocessing (CBP) still relies on emerging engineered microbial consortia that are not yet industrially robust. Future research must focus on integrating pretreatment with these advanced bioprocessing steps to reduce capital costs, minimize handling, and overcome product inhibition (Woźniak et al. 2025; Saritpongteeraka et al. 2020; Chauhan et al. 2024; Liu et al. 2021a; Shahab et al. 2018; Atitallah et al. 2022). The need also arises for novel reactor engineering. The current state of the art for MWP relies heavily on small-scale batch reactors fundamentally limited by microwave penetration depth. For technologies like MWP to transition to pilot-scale, future work must pivot from fundamental chemistry to applied mechanical and chemical engineering, specifically focusing on the design and demonstration of scalable, continuous-flow reactors.

Smart biorefineries and predictive modeling will also be central to efficient lignocellulosic biomass processing. The future lies in combining rapid sensing with real-time predictive ML models capable of optimizing pretreatment severity on the basis of incoming feedstock characteristics. Currently, the state of the art in modeling biorefineries relies heavily on static, steady-state simulations or complex mechanistic kinetic models. While these traditional models are highly effective for baseline techno-economic assessments and plant design, they are computationally intensive and cannot adapt in real-time. Furthermore, while analytical sensors like NIR spectroscopy are available, their integration with dynamic, automated control systems remains in its infancy. Because lignocellulosic feedstock is inherently variable in its composition, applying a one-size-fits-all, static pretreatment condition is highly inefficient. The future lies in the development of smart biorefineries that bridge this gap by transitioning from static simulations to dynamic, real-time predictive models, specifically machine learning, to instantaneously optimize pretreatment severity based on incoming feedstock analysis (Namboonlue et al. 2025; Li et al. 2018; Mais, et al. 2002; Rouabhia et al. 2025). This would maximize sugar yields while minimizing the formation of inhibitors, adapting the process on the fly.

Finally, although lignin-first approaches, such as reductive catalytic fractionation (RCF), have proven highly effective at laboratory scale, they still face major barriers associated with solvent-recovery costs and catalyst deactivation. Research must continue to embrace this full valorization model (Brienza, et al. forthcoming; Makaveckas et al. 2025; Madadi et al. 2025; Ethaib et al. 2020b), by driving a parallel effort in catalysis and bioprocessing to develop industrially robust methods that can convert these complex lignin and hemicellulose structures into a diverse portfolio of value-added products (Gao et al. 2021; Kuo and Lee 2009).

## Data Availability

This manuscript does not report data generation or analysis.

## Abbreviations

AI/ML: Artificial intelligence and machine learning
ASA: Accessible surface area
BM: Ball milling
CapEx: Capital expenditure
CBP: Consolidated bioprocessing
CrI: Crystallinity index
eTEA: Early-stage techno-economic assessment
EtOH: Bioethanol
GWP: Global warming potential:
HMF: 5-Hydroxymethylfurfural
HTTP: Hydrothermal pretreatment
LCA: Life cycle assessment
LCB: Lignocellulosic biomass
LCCs: Lignin-carbohydrate complexes
LHW: Liquid hot water
ML: Machine learning
MSSP: Minimum sugar selling price
MWP: Microwave-assisted pretreatment
NIR: Near-infrared
RCF: Reductive catalytic fractionation
SE: Steam explosion
SF: Severity factor
SSA: Specific surface area
SSF: Simultaneous saccharification and fermentation
TEA: Echno-economic assessment
XOS: Xylooligosaccharides
